# A Comparative Study of Vitrectomy Combined with Internal Limiting Membrane Peeling for the Treatment of Idiopathic Macular Hole with Air or C3F8 Intraocular Tamponade

**DOI:** 10.1155/2018/1672501

**Published:** 2018-07-02

**Authors:** Xiang Chen, Yi Yao, Xiaolu Hao, Xiaocui Liu, Tiecheng Liu

**Affiliations:** Department of Ophthalmology, The Chinese PLA General Hospital, No. 28 Fuxing Road, Haidian District, Beijing 100853, China

## Abstract

**Purpose:**

The treatment of idiopathic macular holes has been basically modeled, and vitreoretinal surgery is recognized as an effective treatment. However, the postoperative tamponade of gas will still make the patient uncomfortable and may have related complications. The purpose of this study is to investigate whether air as an intraocular tamponade is equivalent to gas and what advantages may exist.

**Methods:**

A retrospective study was performed in one hundred and ninety-eight patients from 2013 to 2017; 112 received gas tamponade and 86 received air tamponade. After receiving retinal surgery, the outcomes of best corrected visual acuity, intraocular pressure, slit lamp examination, fundus examination, and imaging of the macula by spectral-domain optical coherence tomography were analyzed.

**Results:**

Before operation, there was no statistically significant difference in age, sex, macular hole diameter, or visual acuity between groups. The median follow-up period for the C3F8 group was 26 months, and the median follow-up for the air group was 25 months. After the operation, the best corrected visual acuity and macular hole closure rate were not significantly different between the two groups. The face-down time after the operation, the incidence of lens opacity on the third postoperative day, the intraocular pressure on the third postoperative day, and the operation time were significantly different between the two groups.

**Conclusions:**

In idiopathic macular hole surgery, the effect of air as an intraocular tamponade material can be similar to that of C3F8 but has fewer complications. In particular, it is a better choice for patients for whom the face-down position is not suitable.

## 1. Introduction

Most macular holes are idiopathic macular holes (IMH), but MH can also be seen in high myopia, trauma, and other situations. The prevalence of IMH is approximately 4/1000 [1] in people over 40 years of age. Among them, 60 to 80 years old is the age with the highest incidence, and it is more commonly seen in women [2, 3]. Although the etiology of IMH is varied and the exact mechanism of the development of IMH remains to be further explored, the consensus has not changed that the principle treatment for MH is vitreoretinal surgery. Currently, the classic procedure for treating IMH is pars plana vitrectomy with peeling of the internal limiting membrane and intraocular gas tamponade, followed by a face-down position for several days [4, 5]. Because of gas tamponade, the face-down position after the IMH surgery can cause much discomfort. It can also cause complicated cataracts, elevated intraocular pressure, secondary glaucoma, and other postoperative complications [6].

Compared to gas, air as an intraocular tamponade has a shorter absorption time in the eye, which means a shorter postoperative face-down time, more comfort for the patient, a lower probability of increased intraocular pressure, and a reduced possibility of concurrent cataracts. However, there are few reports about the use of air for intraocular tamponade: the number of studies is few, and the observed indexes are not comprehensive. It has been reported in the literature that air tamponade is equivalent to long-effect gas filling [7–10] and that the air tamponade effectiveness is poor [11].

Therefore, this study retrospectively analyzed the data of patients undergoing vitrectomy for idiopathic macular hole to investigate whether air as an intraocular tamponade is equivalent to gas and what advantages exist.

## 2. Patients and Methods

### 2.1. Study Design and Patients

Patients were included who consulted the Chinese PLA General Hospital between January 2013 and May 2017, underwent transconjunctival 25-gauge pars plana vitrectomy for the treatment of an idiopathic macular hole, and were followed up for 6 months or longer. All patients gave their written informed consent before participating in the study. No agreement from the ethical committee was needed as only standard procedures were performed.

This study consists of a retrospective evaluation of anatomical and functional results of idiopathic macular hole patients. A total of 198 eyes (46 male and 152 female) from 198 patients (46 men and 152 women) aged 38–80 years (average age of 60 years) were identified.

The inclusion criteria were IMH receiving vitrectomy combined with internal limiting membrane peeling. The exclusion criteria were ocular trauma, high myopia (>6 diopters), optic neuropathy, previous vitreoretinal surgery, and other diseases that may affect visual function.

### 2.2. Surgical Method

All surgeries were carried out under retrobulbar anesthesia. All patients underwent 25-gauge pars plana vitrectomy by a single surgeon. The surgery consisted of a standard 3-port transconjunctival 25-gauge pars plana vitrectomy with triamcinolone-assisted induction of posterior hyaloid separation, and core vitrectomy was performed. Phacoemulsification with intraocular lens implantation was performed simultaneously in 21 of 22 eyes (95.5%). After visualization using indocyanine green, peeling of the inner limiting membrane (ILM) was performed. Finally, a fluid-air exchange was performed. Intraocular tamponade with air or 15% C3F8 was employed at the end of the intervention.

### 2.3. Main Outcome Measures

All patients underwent preoperative and postoperative ophthalmic examinations. Best corrected visual acuity (BCVA) was measured and converted to the logarithm of the minimum angle of resolution (logMAR) scale. Measurements of intraocular tension were carried out using applanation tonometry, evaluation of the anterior segment was by slit-lamp, and examination of the posterior pole was by indirect ophthalmoscopy. The structure of the macular region was evaluated by spectral-domain optical coherence tomography (SD-OCT).

### 2.4. Statistical Analysis

The BCVA results were converted to logMAR equivalents. Statistical analysis was performed using Fisher's exact test or unpaired *t*-test. A *P* value ≤ 0.05 was considered statistically significant. The statistical analyses were performed with SPSS statistics, software version 23.0 (SPSS Inc., Chicago, IL).

## 3. Results

A total of 198 patients were included in the study. The air tamponade group had 86 patients, and the C3F8 group had 112 patients. The average age of the air group was 62.05 years, and the average age of the C3F8 group was 57.88 years, *P*=0.4013. The gender ratios (male/female) were 20/66 and 26/86, *P*=0.8706. Mean macular hole diameters were 333.800 and 403.625, *P*=0.4263, and mean macular hole basal diameters were 665.40 and 873.11 microns, *P*=0.4448. Preoperative mean logMAR visual acuity scores were 0.86 and 0.96, *P*=0.6516, and mean preoperative intraocular pressures were 14.80 and 15.28 mmHg, *P*=0.7783 (Table 1).

All patients were classified according to the diameter of macular hole and divided into 3 groups: <250 microns, 250–400 microns, and >400 microns. The constituent ratios of these three groups were not significantly different, and the *P* values were 0.5223, 0.4440, and 0.8021 (Table 2).

The postoperative follow-up times for the two groups were 25.22 months and 26.37 months, *P*=0.3722, and postoperative logMAR visual acuity scores were 0.520 and 0.387, *P*=0.5678. The face-down times were 6.05 days and 17.98 days, *P*=0.001. On the third day after the operation, the incidence of lens opacity was 17/86 and 58/112, *P*=0.001, intraocular pressures on the third postoperative day were 12.6 and 22.64, *P*=0.0375, and the operation times were 35.07 and 41.63, *P*=0.0006 (Table 3).

**Figure 1:**
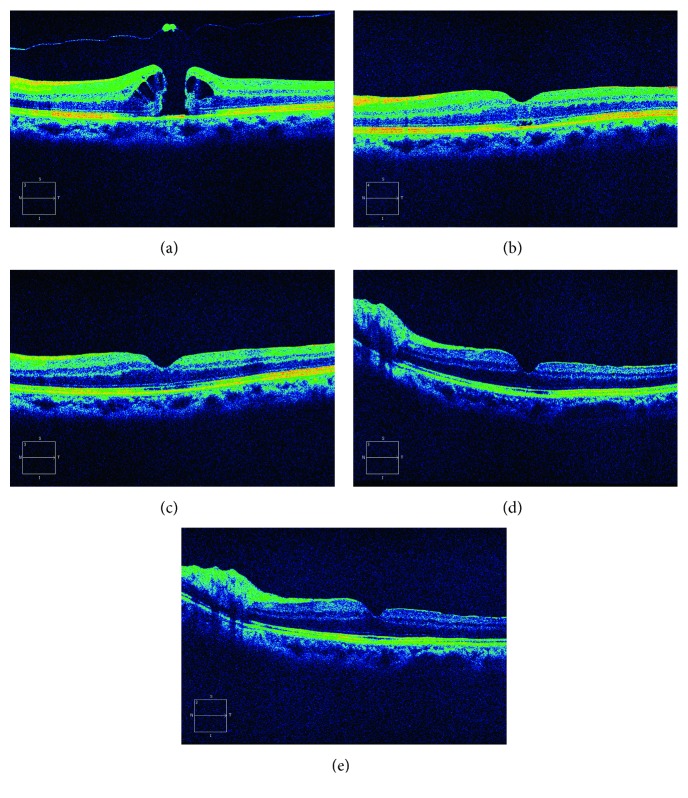
Images from SD-OCT of a 58-year-old woman with a 333 *μ*m diameter MH and a photoreceptor layer defect of 1433 *μ*m in the air group. MH was repaired 3 days after the operation. The ellipsoid zone was restructured at month 3. (a) Preoperative; (b) 3 days after surgery; (c) 1 month after surgery; (d) 3 months after surgery; (e) 6 months after surgery.

**Figure 2:**
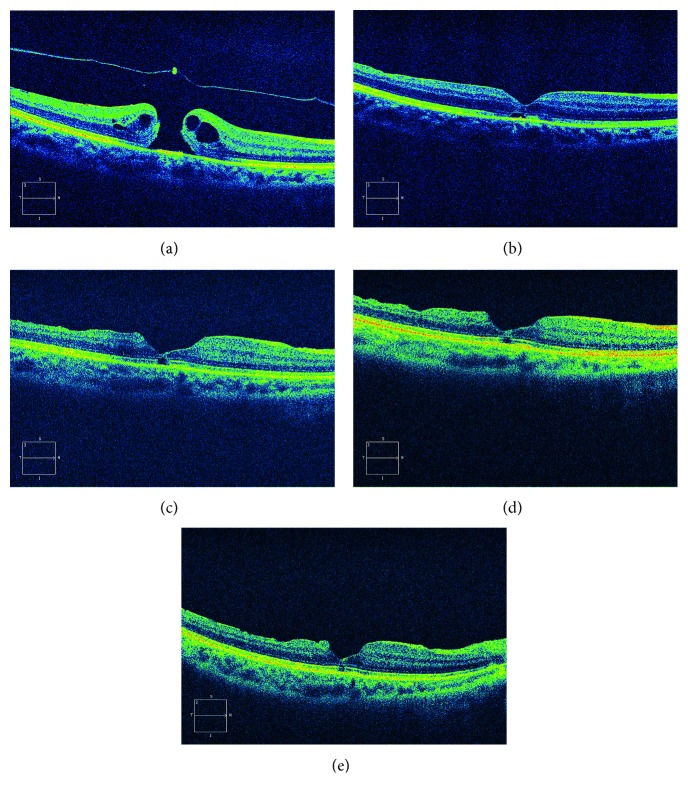
Images from SD-OCT of a 60-year-old woman with a 371 *μ*m diameter MH and a photoreceptor layer defect of 692 *μ*m in the C3F8 group. MH was repaired 3 days after the operation. The ellipsoid zone was not restructured at month 6 with a defect of 606 *μ*m. (a) Preoperative; (b) 3 days after surgery; (c) 1 month after surgery; (d) 3 months after surgery; (e) 6 months after surgery.

The structure of the macular area was examined by OCT before and after operation. The results suggest that the macular hole closure rates were 91.8% and 91.0%, with no significant difference between the two groups (*P*=0.8443). The outer segment layer reconstruction rates at final postoperative follow-up time were 50/86 and 71/112, *P*=0.7704, with no significant difference between the two groups (Table 4, Figures 1 and 2).

## 4. Discussion

Before 1991, scholars thought that IMH was incurable. With a deep understanding of the pathogenesis of idiopathic macular holes, vitreomacular traction is considered to be the most important factor. In 1991, Kelly and Wendel first reported vitreoretinal surgery for IMH [12]. At present, the standard treatment for idiopathic macular hole is vitrectomy combined with ILM peeling, gas filling, and postoperative face down positioning. For a long time, the industry focused on whether the IMH surgery required stripping of the ILM and, during the procedure of stripping the ILM, whether there is a need for the use of stains due to their toxicity. After improving our understanding of macular interface disease, the treatment of IMH has become increasingly more precise.

For an aperture size of less than 250 microns in IMH, intravitreal injection of ocriplasmin may prevent some patients from requiring surgical treatment [13]. For large IMH, the ILM flap reversal technique is often used [14]. The face-down position after the IMH surgery may contribute to patients' discomfort. Lange et al. conducted a randomized controlled study of 30 patients with IMH with a diameter of <400 microns and suggested that the face-down position is not necessary [15]. Of course, the reliability of single-center research is not enough, and a multicenter, large sample study of the face-down position is needed.

Sulfur hexafluoride (SF6), octafluoropropane (C3F8), air, and silicone oil are the most common intraocular tamponade materials. The most widely used tamponade materials is gas, which means SF6 or C3F8. There were some reports of exploratory research on tamponade materials after MH surgery. There are several reports describing using air as the tamponade material after MH surgery, and the conclusion is somewhat controversial. Some doctors reported that air filling is equivalent to gas filling [7–10], but Gesser has reported that the effectiveness of air filling is poor [11].

Compared to the patients who underwent MH surgery with gas, the time in the prone position with air tamponade was shorter, and the patient was more comfortable. The volume of air does not expand, so the probability of elevated intraocular pressure decreases, and the possibility of concurrent cataracts can be reduced. Air filling has many advantages. Therefore, we carried out a study about whether air is a good intraocular tamponade material.

Previous studies have suggested that the stage of MH, duration of symptoms, preoperative visual acuity, size of the MH, and OCT image are predictive factors relevant to postoperative outcome. The most sensitive index for evaluating the recovery of visual function after macular hole surgery is the diameter of the hole before the operation [16, 17]. There were no statistically significant differences between the two groups we chose in terms of patient age, sex, or macular hole diameter, which was an important factor in ensuring the reliability of the study.

The closure of the macular hole requires two important elements. The first is the movement of the traction of the vitreoretinal interface, and the second is the dry environment of the macular interface, which can be achieved by gas or air filling [18, 19]. The blocking effect of the bubble on the hole breaks the fluid from the vitreous cavity into the subretinal space and restricts the cell composition and growth factor from entering the subretinal space.

According to the study by He et al., most of the macular holes of air tamponade eyes can be seen as closed by OCT images 48–72 h after surgery. In addition, the closure time of the macular hole was less than 24 h in SF6 tamponade eyes [10]. Our study showed that most macular holes were closed by the third day after surgery, and the closure rate of air group was 91.8%, while the closure rate of C3F8 group was 91.0%. There was no difference in the closure rate between the two groups, indicating that air tamponade was equivalent to gas tamponade.

There are few articles comparing the effects of air and gas on idiopathic macular hole surgery. Researchers [8] reported that the use of air tamponade for idiopathic macular hole surgery had an equal effect compared with the SF6 group, the postoperative best corrected visual acuity and macular hole closure rate were not significantly different, and the air tamponade group had a shorter face-down time after the operation. Usui et al. [9] reported that the air and SF6 groups had the same macular hole closure rate, postoperative face-down time was different, and the IS/OS layer restructuring rate was not significantly different, which was confirmed by OCT examination. They drew the same conclusion we found here. We observed that there was no difference between the two groups in best corrected visual acuity or macular hole closure rate. The best corrected visual acuity was 0.520 and 0.387, respectively, and the rate of hiatus closure was as previously mentioned. The junction between the IS/OS of the photoreceptors seems to play an important role in the final BCVA. Preoperative reorganization of the IS/OS line is probably achieved by gradual migration of the photoreceptor cells from the surrounding healthy area. There was no significant difference in the length or defect rate of the photoreceptor outer layer between the two groups after the surgery on the photoreceptor outer layer.

The postoperative face-down time in the air tamponade group was 6.05 ± 0.912 days, and the postoperative face-down time in the C3F8 tamponade group was 17.98 ± 3.320 days. The difference between the two groups was statistically significant, and the patients with air tamponade had a shorter face-down time and were more comfortable. We found that on the third day after surgery, there was a higher rate of transient lens opacity in the C3F8 group, which reached 58/112, while the air group was significantly lower, only 17/86. This difference between the two groups was statistically significant. The intraocular pressure (IOP) after the operation was also recorded. On the third day after the operation, the IOP of the C3F8 tamponade group was 22.635 ± 10.78 mmHg, which was significantly higher than that of the air tamponade group at 12.6 ± 2.88 mmHg. Moreover, the operation time for the air tamponade group was shorter than that of the gas tamponade group, and there was a statistically significant difference between the two groups.

The reason for lens opacification may be due to contact and the compression effect of intraocular gas in the posterior capsule of the lens and gas that blocks the metabolic pathway of the lens. We observed that with intravitreal gas absorption, the lens turns transparent again and only a few lenses cannot be restored to transparent; the pathogenesis for this state of permanent opacity needs further study [20]. The causes of ocular hypertension after vitrectomy are varied [21]. It may contribute to laser photocoagulation, combined cataract surgery, severity of postoperative vitreous hemorrhage, and use of expanding gas tamponade. The reason for transient high intraocular pressure after the operation may be the relation to the expansion of C3F8 in our results, because maximal expansion of C3F8 occurred 48–72 hours postoperatively. This time, the interval overlapped with the time of postoperative intraocular hypertension.

Fewer surgical procedures and shorter operation time will reduce the probability of postoperative complications [22]. The main reason for the shorter operation time is the omission of intraocular long-acting gas injection, as the completion of gas fluid exchange is sufficient [23].

In conclusion, the present study revealed air filling has the same therapeutic effect as C3F8 filling, and there is no difference between the macular hole closure rate and the recovery of vision. Additionally, the face-down time is shorter after the operation. Furthermore, we reported that the probability of opacification of the lens and the IOP after the operation was reduced in the air group, and the operation time was shorter. These advantages can make the patient more comfortable.

## Figures and Tables

**Table 1:** Patient characteristics.

	Air group	C3F8 group	*P*
Age (SD)	62.05 (5.788)	57.88 (7.289)	0.4013
Sex, M/F	20/66	26/86	0.8706
Minimum diameter of MH, *μ*m (SD)	333.8 (148.041)	403.625 (148.041)	0.4263
Basal diameter of MH, *μ*m (SD)	665.4 (437.950)	873.111 (488.687)	0.4458
Preoperative mean logMAR VA (SD)	0.86 (0.241)	0.963 (0.451)	0.6516
Preoperative IOP, mmHg	14.8 (3.701)	15.286 (2.138)	0.7783

M, male; F, female; MH, macular hole; logMAR, logarithm of minimal angle resolution; VA, visual acuity; IOP, intraocular pressure.

**Table 2:** The choice of intraocular tamponade material (classification by the diameter of MH).

Diameter of MH, *μ*m	Air group	C3F8 group	*P*
<250	16/86	25/112	0.5223
250–400	40/86	46/112	0.4440
>400	30/86	41/112	0.8021

MH, macular hole.

**Table 3:** Clinical details of patients after surgery.

	Air group	C3F8 group	*P*
Follow-up time, months (SD)	25.219 (9.085)	26.326 (8.437)	0.3772
Prone posturing period, days (SD)	6.05 (0.912)	17.98 (3.320)	0.0005
Opacity of the lens, third day after operation	17/86	58/112	0.0003
Postoperative logMAR visual acuity (SD)	0.520 (0.497)	0.387 (0.340)	0.5678
Operation time, minutes (SD)	35.07 (9.21)	41.63 (10.32)	0.0006
Intraocular pressure, third day after operation, mmHg (SD)	12.6 (2.88)	22.635 (10.78)	0.0375

LogMAR, logarithm of minimal angle resolution.

**Table 4:** Postoperative OCT results.

	Air group	C3F8 group	*P*
Macular hole closure rate (%)	91.8%	91.0%	0.8443
Ellipsoid zone defect diameter, *μ*m (SD)	1306.5 (960.958)	335.750 (325.696)	0.1146
Ellipsoid zone restructuring rate (%)	58.1%	63.4%	0.7704

## Data Availability

The data used to support the findings of this study are included within the article.

## Additional Points

*Limitations of the Study.* The limitations of this study are that it is only a retrospective study at a single-center study, and further research is needed.

## References

[1] Meuer S. M., Myers C. E., Klein B. E. (2015). The epidemiology of vitreoretinal interface abnormalities as detected by spectral-domain optical coherence tomography: the beaver dam eye study. *Ophthalmology*.

[2] Casuso L. A., Scott I. U., Flynn H. W. (2001). Long-term follow-up of unoperated macular holes. *Ophthalmology*.

[3] Kim J. W., Freeman W. R., el-Haig W., Maguire A. M., Arevalo J. F., Azen S. P. (1995). Baseline characteristics, natural history, and risk factors to progression in eyes with stage 2 macular holes. Results from a prospective randomized clinical trial. Vitrectomy for Macular Hole Study Group. *Ophthalmology*.

[4] Parravano M., Giansanti F., Eandi C. M., Yap Y. C., Rizzo S., Virgili G. (2015). Vitrectomy for idiopathic macular hole. *Cochrane Database of Systematic Reviews*.

[5] Thinda S., Shah R. J., Kim S. J. (2015). Two-year anatomical and functional outcomes after macular hole surgery: a prospective, controlled study. *Ophthalmic Surgery, Lasers & Imaging Retina*.

[6] Hasegawa Y., Okamoto F., Sugiura Y., Okamoto Y., Hiraoka T., Oshika T. (2014). Intraocular pressure elevation after vitrectomy for various vitreoretinal disorders. *European Journal of Ophthalmology*.

[7] Hejsek L., Stepanov A., Dusova J. (2017). Microincision 25G pars plana vitrectomy with peeling of the inner limiting membrane and air tamponade in idiopathic macular hole. *European Journal of Ophthalmology*.

[8] Hasegawa Y., Hata Y., Mochizuki Y. (2009). Equivalent tamponade by room air as compared with SF(6) after macular hole surgery. *Graefe’s Archive for Clinical and Experimental Ophthalmology*.

[9] Usui H., Yasukawa T., Hirano Y., Morita H., Yoshida M., Ogura Y. (2013). Comparative study of the effects of room air and sulfur hexafluoride gas tamponade on functional and morphological recovery after macular hole surgery: a retrospective study. *Ophthalmic Research*.

[10] He F., Dong F., Yu W., Dai R. (2015). Recovery of photoreceptor layer on spectral-domain optical coherence tomography after vitreous surgery combined with air tamponade in chronic idiopathic macular hole. *Ophthalmic Surgery, Lasers & Imaging Retina*.

[11] Gesser C., Eckert T., Eckardt U., Porkert U., Eckardt C. (2010). Macular hole surgery with air tamponade. Does air suffice for short-term tamponade?. *Der Ophthalmologe: Zeitschrift der Deutschen Ophthalmologischen Gesellschaft*.

[12] Kelly N. E., Wendel R. T. (1991). Vitreous surgery for idiopathic macular holes: results of a pilot study. *Archives of Ophthalmology*.

[13] Lescrauwaet B., Duchateau L., Verstraeten T., Jackson T. L. (2017). Visual function response to ocriplasmin for the treatment of vitreomacular traction and macular hole: the OASIS study. *Investigative Ophthalmology & Visual Science*.

[14] Duker J. S., Kaiser P. K., Binder S. (2013). The International Vitreomacular Traction Study Group classification of vitreomacular adhesion, traction, and macular hole. *Ophthalmology*.

[15] Lange C. A., Membrey L., Ahmad N. (2012). Pilot randomised controlled trial of face-down positioning following macular hole surgery. *Eye*.

[16] Beausencourt E., Elsner A. E., Hartnett M. E., Trempe C. L. (1997). Quantitative analysis of macular holes with scanning laser tomography. *Ophthalmology*.

[17] Byhr E., Lindblom B. (1998). Preoperative measurements of macular hole with scanning laser ophthalmoscopy. Correlation with functional outcome. *Acta Ophthalmologica Scandinavica*.

[18] Shiode Y., Morizane Y., Matoba R. (2017). The role of inverted internal limiting membrane flap in macular hole closure. *Investigative Ophthalmology & Visual Science*.

[19] Boninska K., Nawrocki J., Michalewska Z. (2017). Mechanism of “flap closure” after the inverted internal limiting membrane flap technique. *Retina*.

[20] Schaefer H., Al Dwairi R., Singh P., Ohrloff C., Kohnen T., Koch F. (2015). Can postoperative accelerated lens opacification be limited by lying in “face-down position” after vitrectomy with gas as tamponade?. *Klinische Monatsblatter fur Augenheilkunde*.

[21] Miele A., Govetto A., Fumagalli C. (2017). Ocular hypertension and glaucoma following vitrectomy: a systematic review. *Retina*.

[22] Naruse S., Shimada H., Mori R. (2017). 27-gauge and 25-gauge vitrectomy day surgery for idiopathic epiretinal membrane. *BMC Ophthalmology*.

[23] Cheng L., Azen S. P., El-Bradey M. H. (2001). Duration of vitrectomy and postoperative cataract in the vitrectomy for macular hole study. *American Journal of Ophthalmology*.

